# Mapping intracellular NAD content in entire human brain using phosphorus-31 MR spectroscopic imaging at 7 Tesla

**DOI:** 10.3389/fnins.2024.1389111

**Published:** 2024-06-07

**Authors:** Rong Guo, Shaolin Yang, Hannes M. Wiesner, Yudu Li, Yibo Zhao, Zhi-Pei Liang, Wei Chen, Xiao-Hong Zhu

**Affiliations:** ^1^Beckman Institute of Advanced Science and Technology, University of Illinois at Urbana-Champaign, Urbana, IL, United States; ^2^Siemens Medical Solutions USA, Inc., Urbana, IL, United States; ^3^Department of Psychiatry, University of Pittsburgh, Pittsburgh, PA, United States; ^4^Department of Bioengineering, University of Pittsburgh, Pittsburgh, PA, United States; ^5^Department of Radiology, Center for Magnetic Resonance Research, University of Minnesota, Minneapolis, MN, United States; ^6^Department of Electrical and Computer Engineering, University of Illinois at Urbana-Champaign, Urbana, IL, United States

**Keywords:** brain metabolites, nicotinamide adenine dinucleotide, phosphorus-31 magnetic resonance spectroscopic imaging, subspace modeling, ultrahigh field

## Abstract

**Introduction:**

Nicotinamide adenine dinucleotide (NAD) is a crucial molecule in cellular metabolism and signaling. Mapping intracellular NAD content of human brain has long been of interest. However, the sub-millimolar level of cerebral NAD concentration poses significant challenges for *in vivo* measurement and imaging.

**Methods:**

In this study, we demonstrated the feasibility of non-invasively mapping NAD contents in entire human brain by employing a phosphorus-31 magnetic resonance spectroscopic imaging (^31^P-MRSI)-based NAD assay at ultrahigh field (7 Tesla), in combination with a probabilistic subspace-based processing method.

**Results:**

The processing method achieved about a 10-fold reduction in noise over raw measurements, resulting in remarkably reduced estimation errors of NAD. Quantified NAD levels, observed at approximately 0.4 mM, exhibited good reproducibility within repeated scans on the same subject and good consistency across subjects in group data (2.3 cc nominal resolution). One set of higher-resolution data (1.0 cc nominal resolution) unveiled potential for assessing tissue metabolic heterogeneity, showing similar NAD distributions in white and gray matter. Preliminary analysis of age dependence suggested that the NAD level decreases with age.

**Discussion:**

These results illustrate favorable outcomes of our first attempt to use ultrahigh field ^31^P-MRSI and advanced processing techniques to generate a whole-brain map of low-concentration intracellular NAD content in the human brain.

## 1 Introduction

Nicotinamide adenine dinucleotide (NAD) is a crucial metabolite for all living cells, existing in the oxidized (NAD^+^) and/or reduced (NADH) forms. It functions as a coenzyme in various cellular redox reactions and serves as a substrate for various NAD^+^-consuming enzymes (Belenky et al., 2007; Verdin, 2015). NAD has been shown to play pivotal roles in numerous cellular processes and functions, including energy metabolism, mitochondrial function, cellular signaling, calcium homeostasis, aging, longevity, and cell death (Ziegler, 2000; Belenky et al., 2007; Verdin, 2015; Cuenoud et al., 2020). Therefore, the *in vivo* measurement and quantification of intracellular NAD levels in the human brain have attracted significant interest in numerous scientific investigations on brain aging (Braidy et al., 2019; Lautrup et al., 2019; Griffiths et al., 2020), cognitive functions (Zhao et al., 2021; Campbell, 2022), and neurodegenerative diseases such as Alzheimer’s (Ruan et al., 2018; Hou et al., 2021; Wang et al., 2021), Parkinson’s (Pérez et al., 2021; Kriebs, 2022; Mischley et al., 2023), and Huntington diseases (Lautrup et al., 2019; Lundt and Ding, 2021).

Despite the profound importance of intracellular NAD metabolism in human health and disease, assessing brain intracellular NAD levels *in-situ* has been challenging. Recent advancements in *in vivo* magnetic resonance (MR) spectroscopy/spectroscopic imaging (MRS/MRSI)-based techniques have enabled non-invasive measurement of NAD levels in the human brain (Lu et al., 2014b; Zhu et al., 2015; Lu et al., 2016; de Graaf et al., 2017; Bagga et al., 2020; Skupienski et al., 2020; Dziadosz et al., 2022). However, most of the current studies were restricted to the examination of a small brain region or using averaged spectra for a few brain regions, with limited exploration of NAD distributions across various brain regions over the entire human brain (Lu et al., 2014b; Zhu et al., 2015; Das et al., 2021; Ren et al., 2023). These limitations stem from several long-standing technical hurdles. The intracellular NAD concentrations in the brain reside within the sub-millimolar range, yielding an exceptionally low signal-to-noise ratio (SNR; Lu et al., 2014b; Zhu et al., 2015). Furthermore, as a high-dimensional imaging method, MRSI typically demands a large number of measurements to encode both spatial and spectral information. Therefore, using existing MRSI methods, especially phosphorus-31 MRSI (^31^P-MRSI) to obtain high-quality maps of NAD concentrations throughout the entire brain could take prohibitively long scan times on the order of hours (Santos-Díaz and Noseworthy, 2020).

In recent years, a range of techniques has been developed to tackle these challenges and advance ^31^P-MRSI applications for mapping brain metabolites. The emergence of ultrahigh field MR systems has yielded significantly enhanced SNR and spectral resolution (Lei et al., 2003; Moser et al., 2012; Barker, 2014; Ruhm et al., 2021). For instance, a 2.8-fold sensitivity increase has been shown in ^31^P-MRS at 7 T compared with 3 T (Rodgers et al., 2014; Lu et al., 2014a). Specialized acquisition methods such as proton decoupling and nuclear overhauser effect (NOE) have been utilized to further improve spectral quality and SNR (Luyten et al., 1989; Lei et al., 2003); and fast scanning trajectories like echo-planar or spiral trajectories have been used to enhance data acquisition speed (Valkovič et al., 2016; Korzowski and Bachert, 2018). Advanced post-processing methods like low-rank denoising have further contributed to noise reduction (Ma et al., 2017; Clifford et al., 2020; Korzowski et al., 2020). Nevertheless, quantitative imaging of the intracellular NAD level throughout the entire human brain still remains challenging given its exceptionally low concentration.

The purpose of this study is to demonstrate the feasibility of whole-brain NAD mapping by synergistically integrating ultrahigh field ^31^P-MRSI with an advanced denoising method based on the probabilistic subspace model (Chen et al., 2020; Li et al., 2021a; Zhang et al., 2023). This method leverages ultrahigh field, spectral priors, spatial constraints, and statistical priors to significantly enhance the sensitivity of ^31^P-MRSI for NAD imaging. Its performance in terms of SNR, accuracy, and reproducibility of the resulting NAD measurements were evaluated. The potential of mapping tissue metabolic heterogeneity and revealing age dependence of NAD levels were also preliminarily assessed. The results indicate that this method holds a significant promise for mapping the NAD content in the human brain.

## 2 Materials and methods

### 2.1 Data acquisition

Two sets of *in vivo* human brain ^31^P-MRSI data were collected in this study. The first set of data was collected at the University of Minnesota for technical validation. The scans were performed on a 90-cm bore 7 T magnet (Magnex Scientific, Abingdon, United Kingdom) equipped with a Varian INOVA console and a ^31^P/^1^H dual-tuned TEM head volume coil. The scan protocol included an anatomical imaging using a turboFLASH sequence (repetition-time (TR)/echo-time (TE)/inversion-time (TI) = 8.0/3.6/1,600 ms, field of view (FOV) = 20 × 20 cm^2^, matrix size = 128 × 128, slice thickness = 3 mm), and 3D ^31^P-MRSI using the chemical shift imaging (CSI) sequence with a Fourier series window imaging technique (Hendrich et al., 1994) (TR/TE = 500/1.0 ms, flip angle = 45°, bandwidth = 5 kHz, FOV = 20 × 20 × 22 cm^3^, matrix size = 15 × 15 × 13, cylindric voxel shape, nominal voxel size = 2.3 cc, total scan time = 51 min). Seven healthy volunteers participated in this study and the scan procedures were approved by the Institutional Review Board of the University of Minnesota. One of the subjects was scanned twice for the test–retest reproducibility study. The second set of data was collected at the University of Pittsburgh, with higher spatial resolutions to investigate the potential on assessing tissue metabolic heterogeneity. These scans (from 14 subjects) were performed on a MAGNETOM 7 T system (Siemens Healthcare, Erlangen, Germany) with a ^31^P/^1^H dual-tuned birdcage head volume coil (Rapid Biomedical, Rimpar, Germany), under approval of the Institutional Review Board at the University of Pittsburgh. The scan protocol included an MPRAGE sequence (TR/TE/TI = 3000/1.87/1,200 ms, FOV = 25.6 × 18.4 cm^2^, matrix size = 256 × 184, slice thickness = 1 mm) and a 3D ^31^P-CSI sequence (TR/TE = 200/1.0 ms, flip angle = 30°, bandwidth = 5 kHz, FOV = 22 × 22 × 10 cm^3^, matrix size = 24 × 24 × 8, cuboid voxel shape, nominal voxel size = 1.0 cc, total scan time = 21 min). In the ^31^P-CSI sequence, two 5-ms hard pulses were applied on the water proton resonance for NOE to gain SNR. A summary of the hardware and acquisition parameters according to the Minimum Reporting Standards for *in vivo* MRS (Lin et al., 2021) was included in Supplementary Tables S1–S3. Informed consent was collected from all the volunteers at both sites before the experiments. These two datasets are referred to as “2.3-cc data” and “1.0-cc data” in the subsequent sections of this paper.

### 2.2 Signal denoising

Existing low-rank denoising methods for ^31^P-MRSI signals exploit the partial separability property of the spatiotemporal distributions (Liang, 2007), which admits the following decomposition (or low dimensional subspace structure) of the noise-free signal (denoted as $\rho \left(x,t\right)$ ) in Equation (1):


(1) $$\rho \left(x,t\right)=\overset{L}{\underset{l=1}{\sum }}u_{l}\left(x\right)v_{l}\left(t\right)\text{,}$$

where $\left\{v_{l}\left(t\right)\right\},\left\{u_{l}\left(x\right)\right\},\;L$ denote the temporal basis functions, corresponding spatial coefficients, and the model order, respectively. This partial separability model implies that the actual degrees-of-freedom needed to represent the MRSI signals can be largely reduced, and the Casorati matrix formed by the MRSI signals has a low-rank structure (Liang, 2007). Based on this property, the typical low-rank denoising methods perform low-rank approximation on the Casorati matrix for noise reduction (Nguyen et al., 2013). First, the Casorati matrix is constructed using the measured spatiotemporal MRSI signals. Then, a singular value decomposition (SVD) is performed on the Casorati matrix. Given a specific model order L , the singular value matrix from SVD is truncated by only keeping the first L values and setting the others to zero. Then the truncated singular value matrix is multiplied back with the other two decomposed matrices to generate the denoised Casorati matrix thus the denoised MRSI signals. Although its effectiveness has been demonstrated in various MRSI applications (Liang, 2007; Lam and Liang, 2014; Ma et al., 2017; Korzowski et al., 2020), it may fall short in addressing the challenges posed by extremely low SNR scenarios, particularly in the context for mapping brain NAD.

In this study, we used a probabilistic subspace model-based method for more effective denoising, which integrated the low-rank property with group spectral priors, spatial and statistical constraints (Li et al., 2021a,b). More specifically, the temporal basis functions $\left\{v_{l}\left(t\right)\right\}$ were pre-determined from a group of data (all the ^31^P-MRSI data we acquired in this study) instead of a single noisy data as spectral priors; the spatial coefficients were assumed to have no large spatial variations and follow certain statistical distribution, which were also pre-estimated from the group data. To incorporate these priors and impose these constraints, the denoising was performed by solving the following regularized optimization problem in Equation (2):


(2) $$\hat{U}=\arg\min_{U}\;\rho_{r}-{UV}_{2}^{2}+\lambda {WU}_{2}^{2}-\sigma_{n}^{2}\mathrm{logPr}\left(U\right)\text{,}$$

where U , V , and $\rho_{r}$ are the matrix forms of $\left\{u_{l}\left(x\right)\right\}$ and $\left\{v_{l}\left(t\right)\right\}$ and measured noisy ^31^P-MRSI data, respectively. W denotes the edge-weighted total variation operator, λ is the weighting parameter (determined by the discrepancy principle (Vogel, 2002)), $\mathrm{Pr}\left(U\right)$ denotes the probability given a specific U , and $\sigma_{n}^{2}$ is the variance of measurement noise. With the estimated $\hat{U}$ , the denoised ^31^P-MRSI signals were generated as $\rho_{d}=\hat{U}V$ .

### 2.3 Subspace and distribution estimation

To pre-determine the basis functions $\left\{v_{l}\left(t\right)\right\}$ , a spectral alignment step was first performed to all the ^31^P-MRSI data in the group. Specifically, the frequency of phosphocreatine (PCr) resonance peak in each voxel was first estimated using Hankel singular value decomposition (HSVD), then this frequency was used to shift the signals so as to align the PCr peak to 0 ppm (Barkhuijsen et al., 1987). This spectral alignment step removed the inter-voxel and inter-scan frequency variations caused by the static magnetic field (B_0_) inhomogeneity, thus promoting the low rankness of ^31^P-MRSI signals (Peng et al., 2010). After the spectral alignment, a Casorati matrix was formed including all the group ^31^P-MRSI data, with rows as the temporal signals of each spatial location. Then, SVD was performed on the Casorati matrix to derive the temporal basis functions (Liang, 2007; Nguyen et al., 2013; Chen et al., 2020; Lam et al., 2020; Guo et al., 2021a,b).

To estimate the statistical distributions $\mathrm{Pr}\left(U\right)$ , the group ^31^P-MRSI data were first projected onto the subspace spanned by the basis functions derived above to generate corresponding spatial coefficients. This set of spatial coefficients formed the empirical distributions of $\left\{u_{l}\left(x\right)\right\}$ . In our current implementation, we used a Gaussian model to express these distributions: $\mathrm{Pr}\left(U\right)=Ae^{-∥U-U_{0}{∥}_{2}^{2}/\sigma_{U}^{2}}$ . A is a normalization constant; the mean ( $U_{0}$ ) and variance ( $\sigma_{U}^{2}$ ) values were derived from the group data in the maximum likelihood sense, as done in the previous works (Boldea and Magnus, 2009; Li et al., 2021a).

### 2.4 Quantification

After denoising, the spectral quantification was performed using a time-domain fitting method to generate the signal intensity of each separate metabolite (Ratiney et al., 2005; Li et al., 2017). The basis set used for spectral fitting included 11 resonance structures of the measurable ^31^P metabolites in the human brain: PCr, α-adenosine triphosphate (αATP), γATP, βATP, (intracellular) inorganic phosphate (Pi), extracellular Pi (ePi), glycerophosphoethanolamine (GPE), glycerophosphocholine (GPC), phosphoethanolamine (PE), phosphocholine (PC), and NAD combining NAD^+^ and NADH. Given the linewidth broadening for the *in vivo* ^31^P-MRSI data and disappearing of doublets or triplets at ultrahigh field of 7 Tesla, the ^31^P signals of these metabolites were modeled as singlet resonances with Lorentzian line shape. After spectral fitting, the quantitative metabolite concentrations were calculated using γATP resonance signal as an internal reference, as in Equation (3):


(3) $$C_{m}=\frac{C_{\gamma ATP}}{N_{m}}\frac{E_{\mathrm{\gamma ATP}}}{E_{m}}\frac{S_{m}}{S_{\mathrm{\gamma ATP}}}$$

where C,S,E,N are the concentration (mM), fitted signals intensity, longitudinal relaxation time (T_1_) saturation factor, and number of ^31^P spins in each metabolite, respectively. $C_{\mathrm{\gamma ATP}}$ was set as 2.8 mM (Zhu et al., 2015). The T_1_ saturation factor was calculated as in Equation (4):


(4) $$E_{m}=\frac{\left(1-e^{-TR/T_{1,m}}\right)\sin\alpha }{1-\cos\alpha e^{-TR/T_{1,m}}}$$

where α is the RF pulse flip angle, and $T_{1,m}$ the longitudinal relaxation time of a metabolite, whose values from the previous reports were used (Ren et al., 2015).

### 2.5 Performance evaluation

Performance of the presented method for NAD mapping was evaluated in multiple aspects. First, using one “2.3-cc data,” the SNR enhancement by the denoising method was calculated and compared with the original measurements and the typical low-rank approximation method. The SNR was calculated as the PCr peak height divided by the noise standard deviation estimated from the last one hundred time points of spectroscopic signals. Mean values and standard deviation of the resulting NAD concentrations were also computed and compared. Second, the accuracy of the NAD measurements was assessed via computational simulation. One high SNR ^31^P-MRSI data was created by averaging the eight “2.3-cc data” as ground truth, and Gaussian noise was added to mimic the actual measurements. Then, NAD concentrations were obtained using the processing methods mentioned above, and the relative root-mean-square-error (rRMSE) with ground truth was quantified. Detailed setup of the simulation was described in the next section. Third, the test–retest “2.3-cc data” was used to evaluate the reproducibility. NAD concentrations obtained from two scans on the same subject were compared; and Pearson’s correlation coefficient as well as coefficient of variation (CoV) were reported. Fourth, the “1.0-cc data” was used to assess the metabolic heterogeneity between the gray and white matter. More specifically, a linear regression analysis was performed on metabolite signal intensities against corresponding gray matter fractions. In this analysis, gray matter fractions were calculated from tissue segments derived from the anatomical image using SPM12 (Penny et al., 2007). Only voxels totally within the brain and with a cerebrospinal fluid (CSF) fraction less than 30% were included. Fifth, the correlation between NAD levels in the “1.0-cc data” and the subject ages were analyzed to assess the age dependence. The average NAD level over the brain was calculated for each subject and a linear regression analysis was performed to show the trend of NAD level changing with their age.

### 2.6 Computational simulation

The generation of ground truth for computational simulation based on real ^31^P-MRSI data included the following steps. First, all the eight “2.3-cc data” were spatially registered together using affine transformation. Second, the spectral alignment step was performed to all the ^31^P-MRSI data to align their PCr peaks to 0 ppm. Third, the phase differences between different ^31^P-MRSI data were estimated using their first temporal points and then they were corrected to match the first data. Fourth, these ^31^P-MRSI data of different subjects were averaged, and one Hamming window was applied in the slice direction of the average ^31^P-MRSI data (in k-space) to gain SNR as the ground truth. After the high SNR ground truth was created, Gaussian noise with the same variance level as in practically measured data was added, generating the noisy raw data. Then, signal denoising and spectral quantification as mentioned above were applied to generate NAD estimates for further evaluation.

## 3 Results

**Figure 1:**
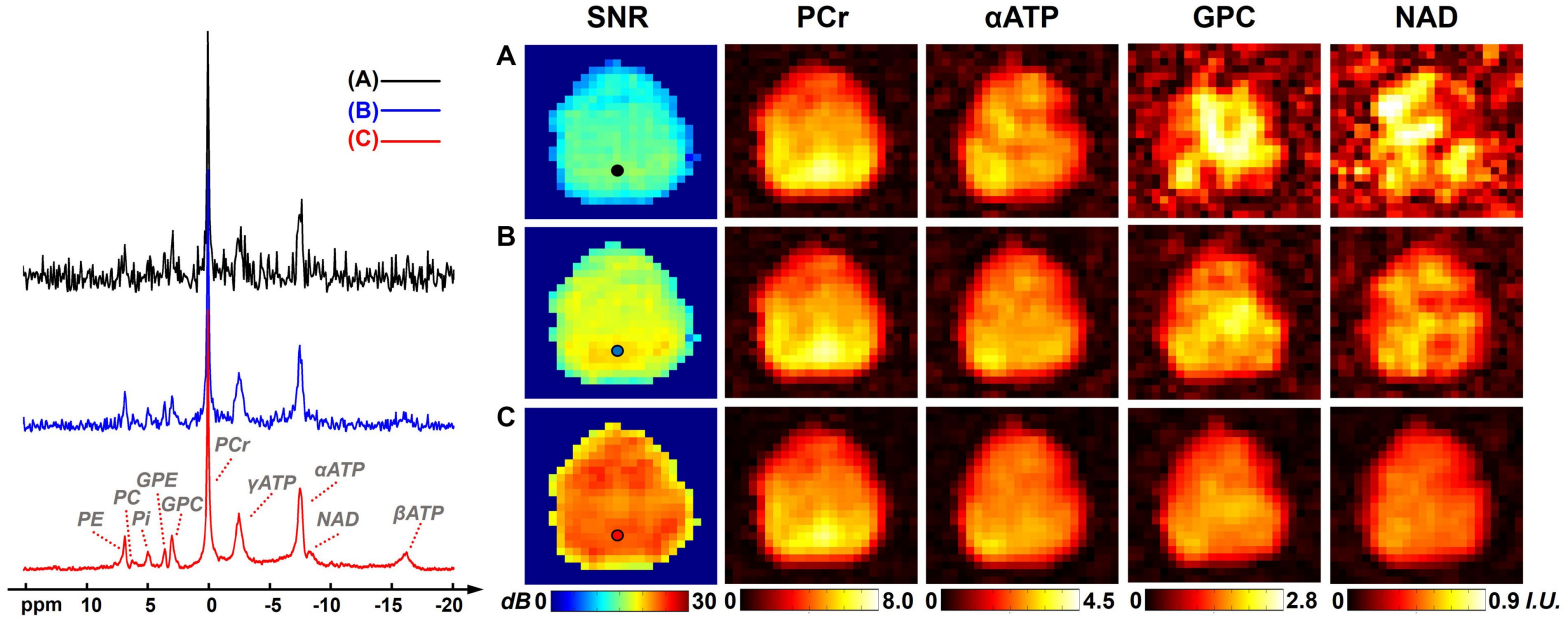
Representative spectra, SNR maps, and metabolite maps (signal intensities after spectral fitting, including PCr, αATP, GPC, and NAD) of results (from one 2.3 cc data) using different processing methods: **(A)** raw *in vivo* ^31^P-MRSI data without denoising; **(B)** ^31^P-MRSI data using basic low-rank denoising; **(C)** ^31^P-MRSI data using the probabilistic subspace-based denoising method. Spectra were displayed on the same horizontal scale. The displayed spectra were from the single voxel labeled on the SNR maps and they were displayed in absolute mode.

Figure 1 shows a comparison of resulting ^31^P-MRSI spectra, SNR maps, and metabolite maps (on one set of “2.3-cc data”) using different processing methods to illustrate the denoising efficacy of the presented method. On the representative spectra from a selected voxel, we can see many metabolite peaks other than PCr and ATPs were buried under the noise level in the raw ^31^P-MRSI data; the basic low-rank denoising method performed well in noise reduction but the NAD peak was still difficult to be distinguished from noise; while the results from the proposed method achieved a better noise reduction and the NAD peak became clearly above the noise level. The SNR maps also affirmed the significant improvement in SNR compared with original data and basic low-rank denoising. The mean SNRs over the brain were 12.65 ± 1.91 dB, 17.05 ± 1.67 dB, and 22.49 ± 1.48 dB for raw MRSI data, low-rank denoising, and the proposed method, respectively. Compared with the original noisy data, the presented method almost provided an around 10-fold sensitivity enhancement, which might make it possible for reliable detection of NAD signals in the voxel base. As shown in Figure 1, given the relatively high concentration of PCr, its spatial intensity maps were of good quality and consistent using different methods. But the spatial maps of low-concentration metabolites like GPC and NAD were heavily contaminated by noise without denoising. The NAD maps using basic low-rank denoising showed significant noise reduction but there were still noticeable spatial variations induced by noise. In contrast, the results produced using the proposed method showed further reduced noise fluctuations and a relatively homogenous distribution of NAD within the human brain. The signal intensities of NAD over the brain were 0.52 ± 0.18, 0.49 ± 0.11, and 0.42 ± 0.08 (in institutional unit) for these three methods, respectively. As expected, the proposed method produced the smallest spatial variations.

**Figure 2:**
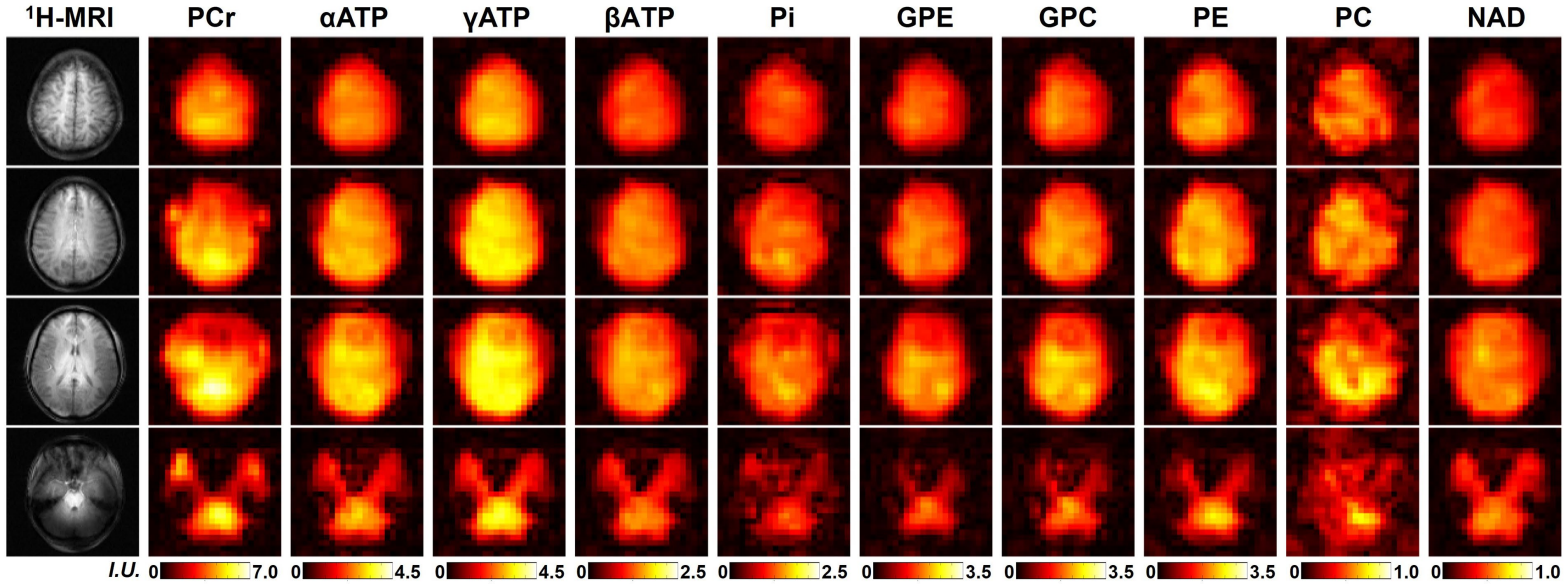
A representative set of human brain metabolite maps (signal intensities after spectral fitting) obtained using the proposed method, including PCr, αATP, γATP, βATP, Pi, GPE, GPC, PE, PC, and NAD. Nominal spatial resolution was 2.3 cc, acquisition time was 51 min.

One complete set of whole-brain metabolite intensity maps (4 representative ^31^P-MRSI slices from another set of “2.3-cc data”) is shown in Figure 2, including PCr, αATP, γATP, βATP, Pi, GPE, GPC, PE, PC, and NAD. Most of the intensity maps, including NAD, were of high quality with very minor effects of noise, except PC, which had the lowest signal intensity in the acquired ^31^P-MRSI data (as shown in the spectra in Figure 1). The NAD map suggested that intracellular NAD concentrations appeared to have a relatively homogeneous distribution over the brain, with only slight enhancements in midbrain or thalamic regions. It is worth noting that PCr had high signals in some subcutaneous regions, but other metabolites did not, which was suspected due to the scalp muscles with a higher PCr concentration.

**Figure 3:**
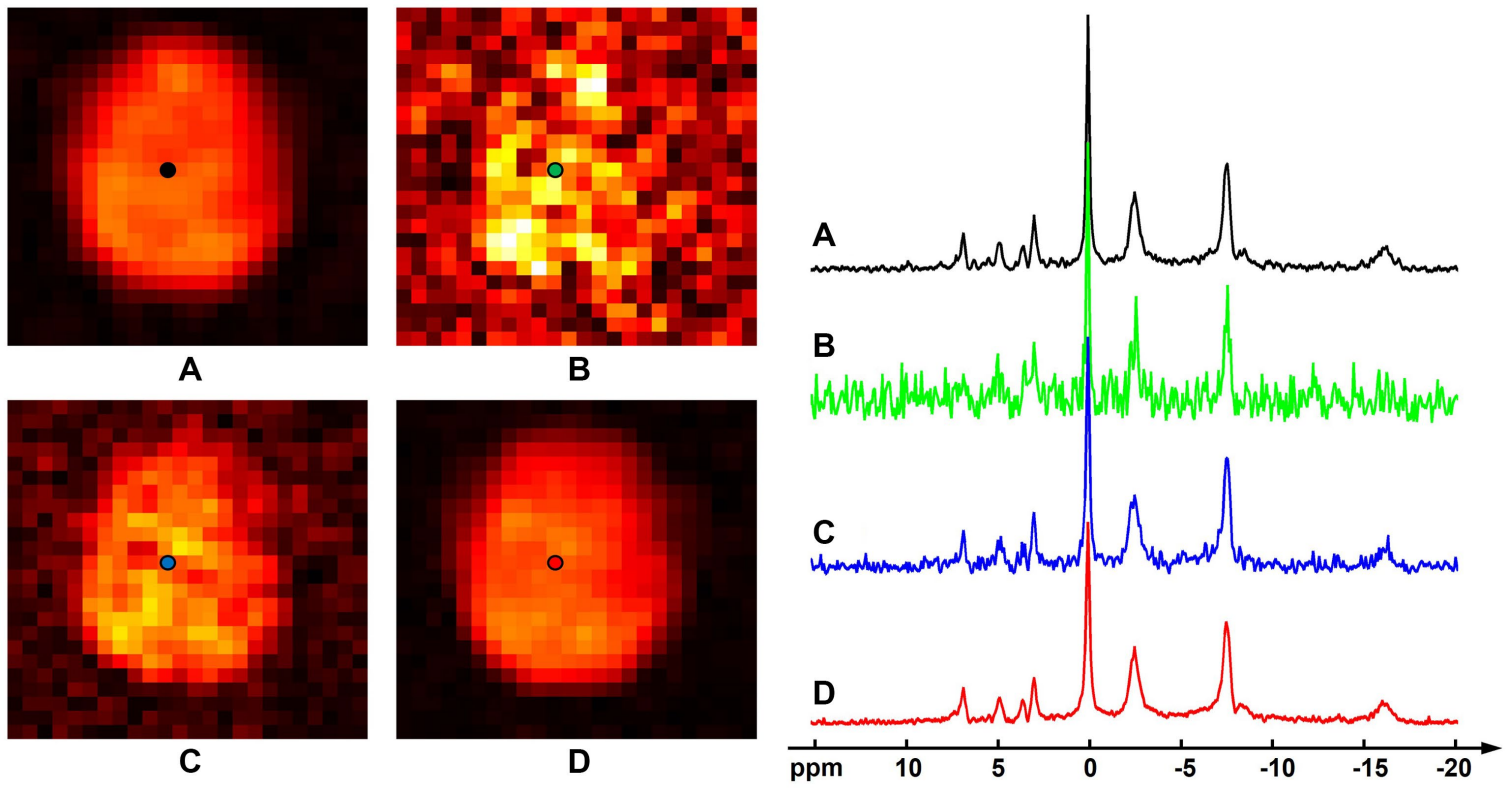
Computational simulation of ^31^P-MRSI for comparison of different methods: **(A)** ground truth (generated by averaging eight ^31^P-MRSI data); **(B)** raw data without denoising; **(C)** basic low-rank denoising; **(D)** probabilistic subspace-based denoising method. NAD maps (signal intensities after spectral fitting) were displayed on the left and the localized spectra of selected point (as labeled on the NAD maps) were displayed on the right. The spectra were displayed in absolute mode.

Figure 3 shows results of the computational simulation to illustrate accuracy of the proposed method. Representative spectra and NAD estimates using different processing methods were compared. Similar to the observations in Figure 1, raw measurements without denoising had very noisy estimates of NAD intensity, the low-rank denoising provided a reduced noise level, while the proposed method offered the best SNR. With a significant noise reduction, the NAD intensity map and localized spectrum produced using the proposed method were very close to the ground truth. Quantitatively, rRMSEs of NAD estimates referring to the ground truth were 48.7, 20.5, and 12.4% for the raw data, low-rank denoising, and the proposed method, respectively. In this realization of noise simulation, SNR enhancement of the proposed method led to an improved accuracy on NAD estimation by around six-fold, and the resulting relative errors were around 12%.

**Figure 4:**
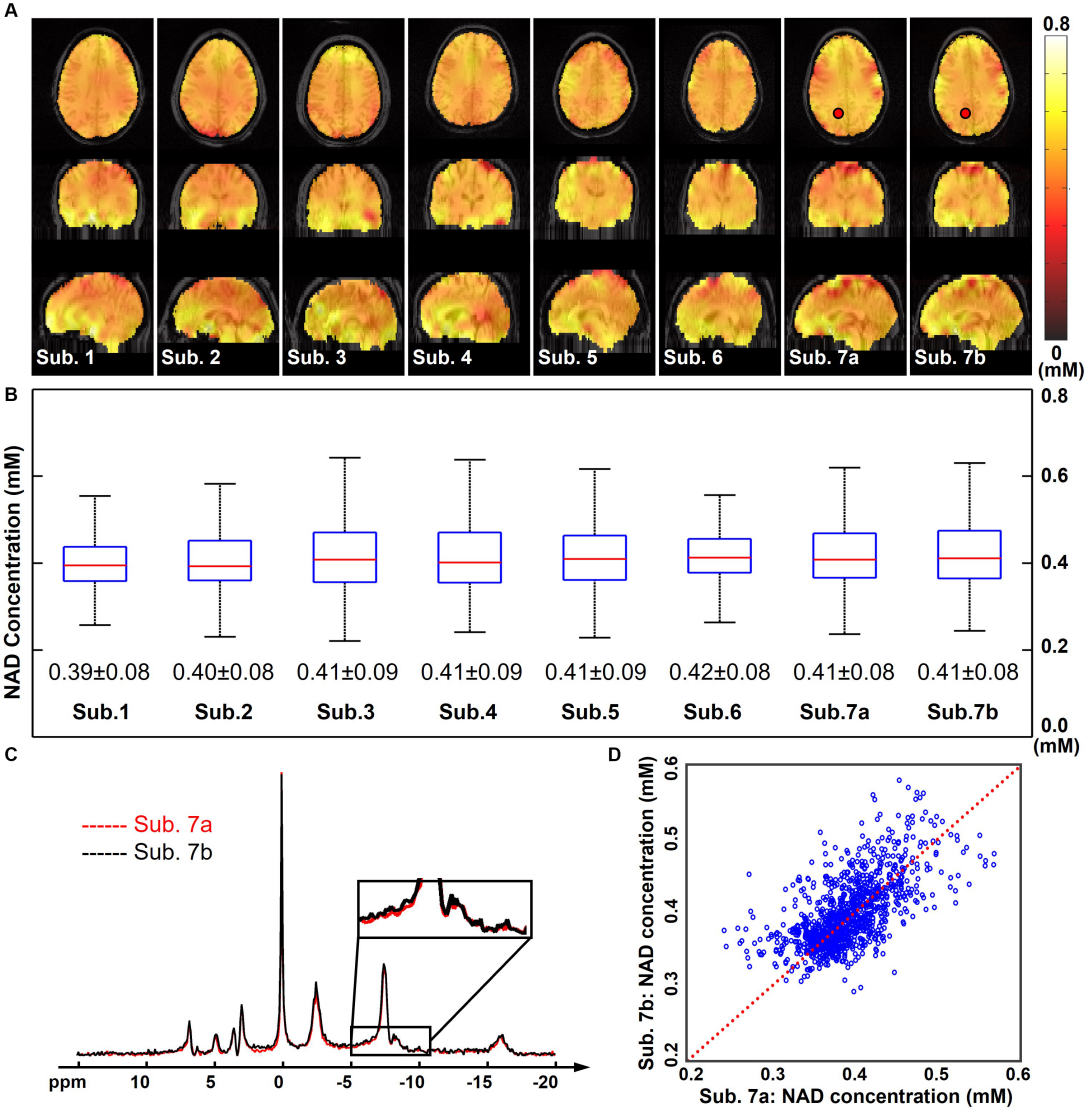
**(A)** Quantified whole-brain NAD concentration maps (in mM, overlaid on anatomical MRI images) of seven subjects (the subject 7 was scanned twice, labeled as Sub. 7a and Sub. 7b). **(B)** Boxplots of the NAD concentrations of these seven subjects with the mean and standard deviations. Red lines indicate the median while blue boxes cover the 25%–75% percentiles. **(C)** Spectra of the same selected voxel (as labeled on the NAD maps) from Sub. 7a and Sub. 7b datasets. **(D)** R2-plot of the NAD concentrations from Sub. 7a and Sub. 7b datasets, with the Pearson’s correlation coefficient as 0.72 and the coefficient of variation as 6.1%. Red dot line is the identical line. For all these scans, nominal spatial resolution was 2.3 cc, acquisition time was 51 min. The spectra were displayed in absolute mode.

Figure 4A displays the quantified NAD concentration maps (in the unit as mM) from all seven subjects in the “2.3-cc data.” These NAD concentrations are displayed in tripolar views and show similar spatial distributions in the brain. A summary of the NAD concentrations across these subjects is shown in Figure 4B. The mean NAD concentration of each subject was around 0.4 mM, and the standard deviation was around 0.1 mM. These statistics were reasonably consistent among these seven subjects studied to date. Figure 4 also includes the reproducibility results from two repeated scans on the same subject. One pair of spectra in the same spatial voxel from these two measurements were displayed and compared in Figure 4C. A good agreement was observed between these two spectra, including the small NAD peaks, as indicated in the zoom-in regions between −5 and −10 ppm. The R2-plot in Figure 4D includes quantitative NAD concentrations of all voxels within the brain. Compared with the identical line, there was no significant bias between these two NAD measurements, and the NAD concentrations were mostly distributed from 0.30 mM to 0.50 mM. Additionally, the Pearson’s correlation coefficient between these two measurements was 0.72 and the averaged coefficient of variation was 6.1%, further suggesting an excellent reproducibility between these two scans, even though the NAD concentration is very low.

**Figure 5:**
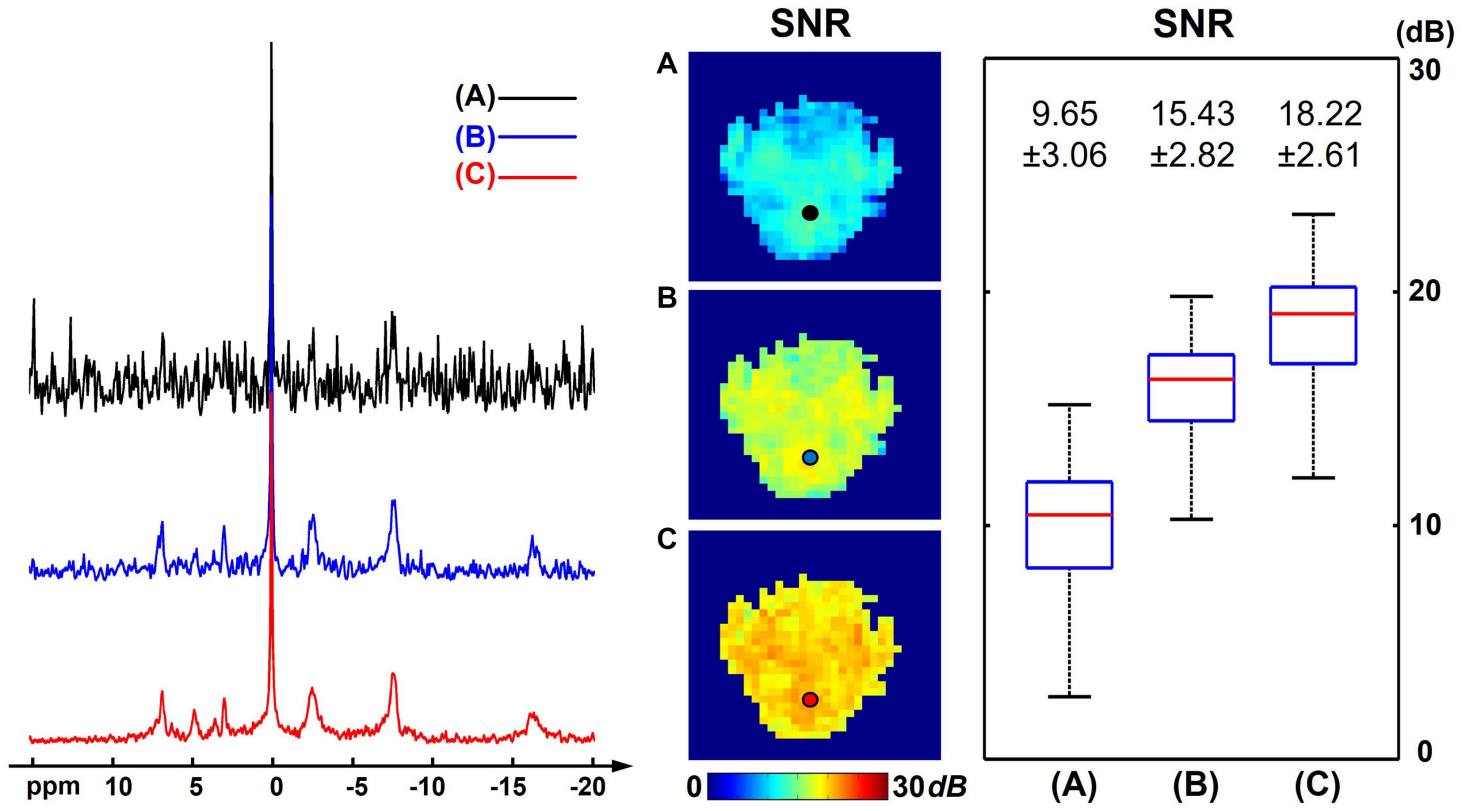
SNR analysis of one high-resolution “1.0-cc data” (age 22, male), including representative spectra, SNR maps, and SNR boxplots of results using different processing methods: **(A)** raw *in vivo* ^31^P-MRSI data without denoising; **(B)** ^31^P-MRSI data using basic low-rank denoising; **(C)** ^31^P-MRSI data using the probabilistic subspace-based denoising method. The displayed spectra were from the single voxel labeled on the SNR maps. The spectra were displayed in absolute mode.

Figure 5 presents the SNR analysis on one high-resolution “1.0-cc data” using different processing methods. Compared with the “2.3-cc data,” this set of data was acquired with higher resolution and a shorter scan time, thus intrinsically having a lower SNR. The SNR differences with the “2.3-cc data” could be noticed from the representative spectra and SNR maps compared with Figure 1. But similarly, the proposed method still outperformed the raw MRSI data and basic low-rank denoising method and produced spectra of a largely improved quality, with the NAD resonance visible above the noise level. The calculated SNRs over the brain were 9.65 ± 3.06 dB and 18.22 ± 2.61 dB before and after denoising, approximately one half and one third of the “2.3-cc data,” respectively.

**Figure 6:**
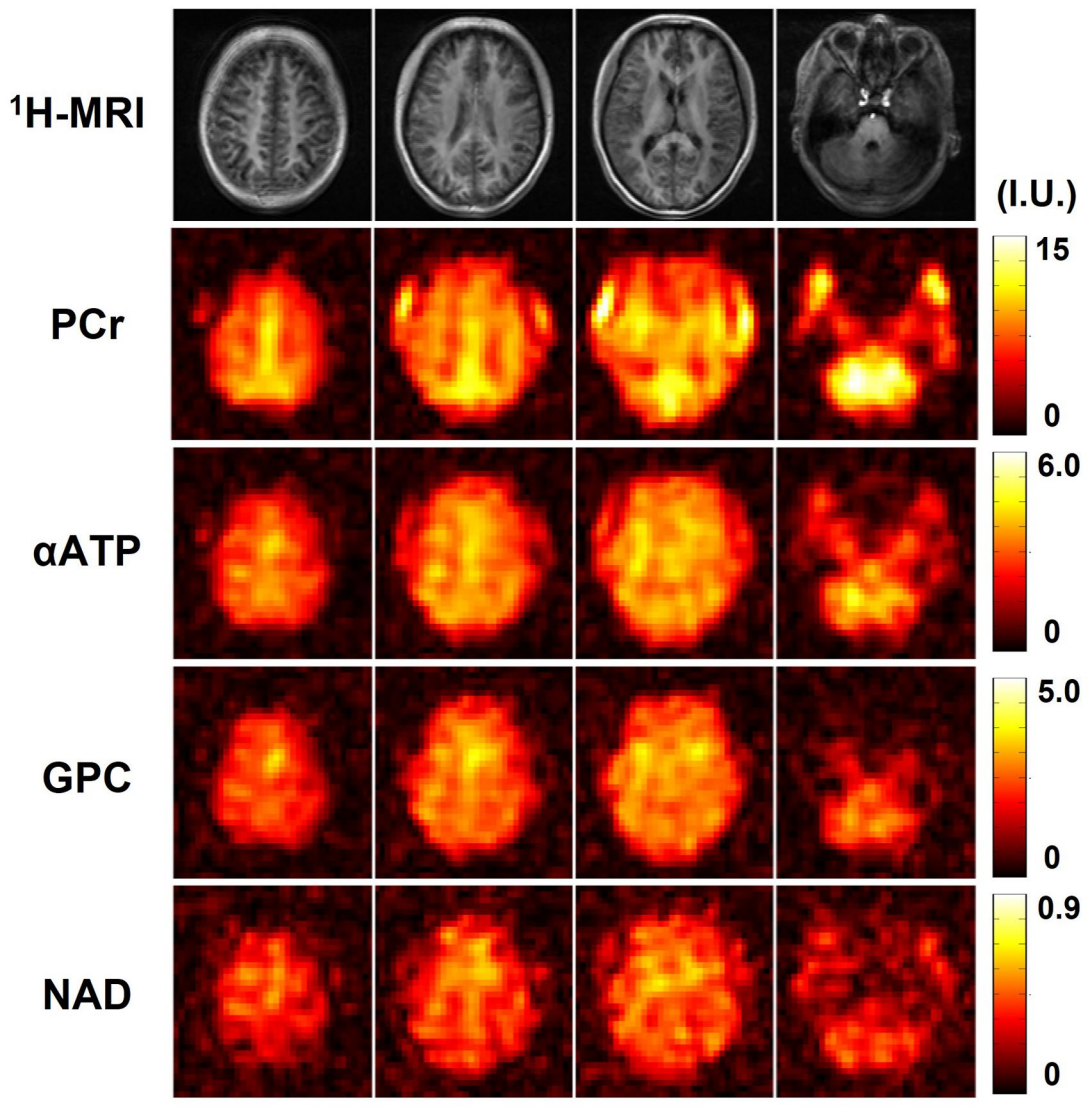
Resulting metabolite maps (signal intensities after spectral fitting, including PCr, αATP, GPC, and NAD) of one high-resolution ^31^P-MRSI data (age 22, male). Nominal spatial resolution was 1.0 cc, acquisition time was 21 min.

**Figure 7:**
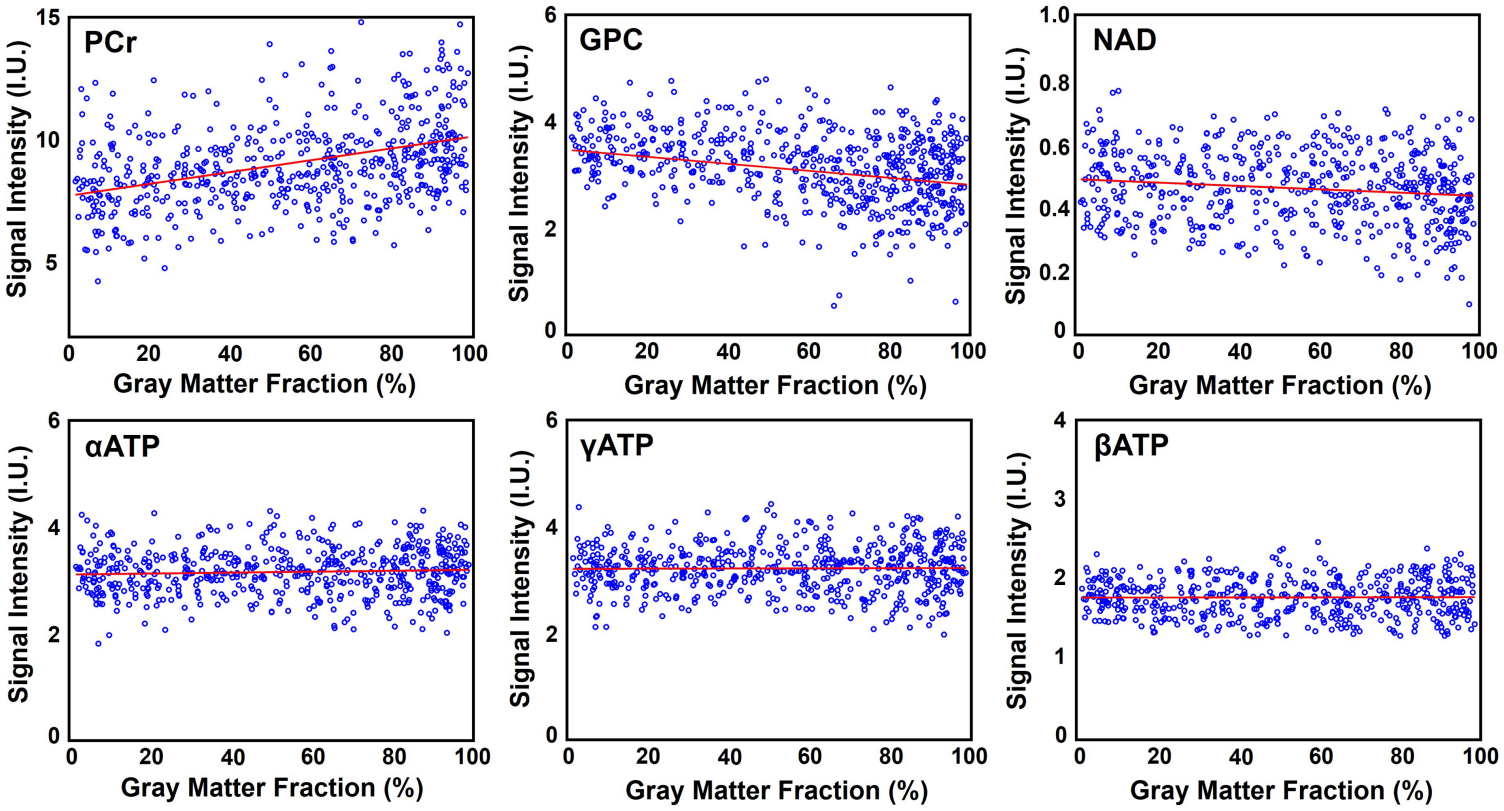
Regression analysis of the metabolite signal intensities over the gray matter fraction (in percentage), including PCr, GPC, NAD, αATP, γATP, and βATP. Red lines are linear regression curves.

Figure 6 shows the high-resolution metabolite maps obtained from one set of “1.0-cc data,” including intensity maps of PCr, αATP, GPC, and NAD. With the degraded SNR compared with the “2.3-cc data,” the proposed method still produced reasonable although relatively noisier NAD estimates. Nevertheless, benefitting from the higher resolution, the heterogeneity of metabolite distributions between brain tissues became more visible. More specifically, PCr exhibited a clear higher concentration in gray matter than white matter, while the ATPs and NAD showed relatively uniform distributions between brain tissues. The regression analysis results of this data are shown in Figure 7. From the plots and regression curves, we found that higher gray matter fractions were associated with higher PCr levels, lower GPC levels, uniform ATP distributions, and similar NAD levels, which matched the observations in literature reports (Ruhm et al., 2021).

**Figure 8:**
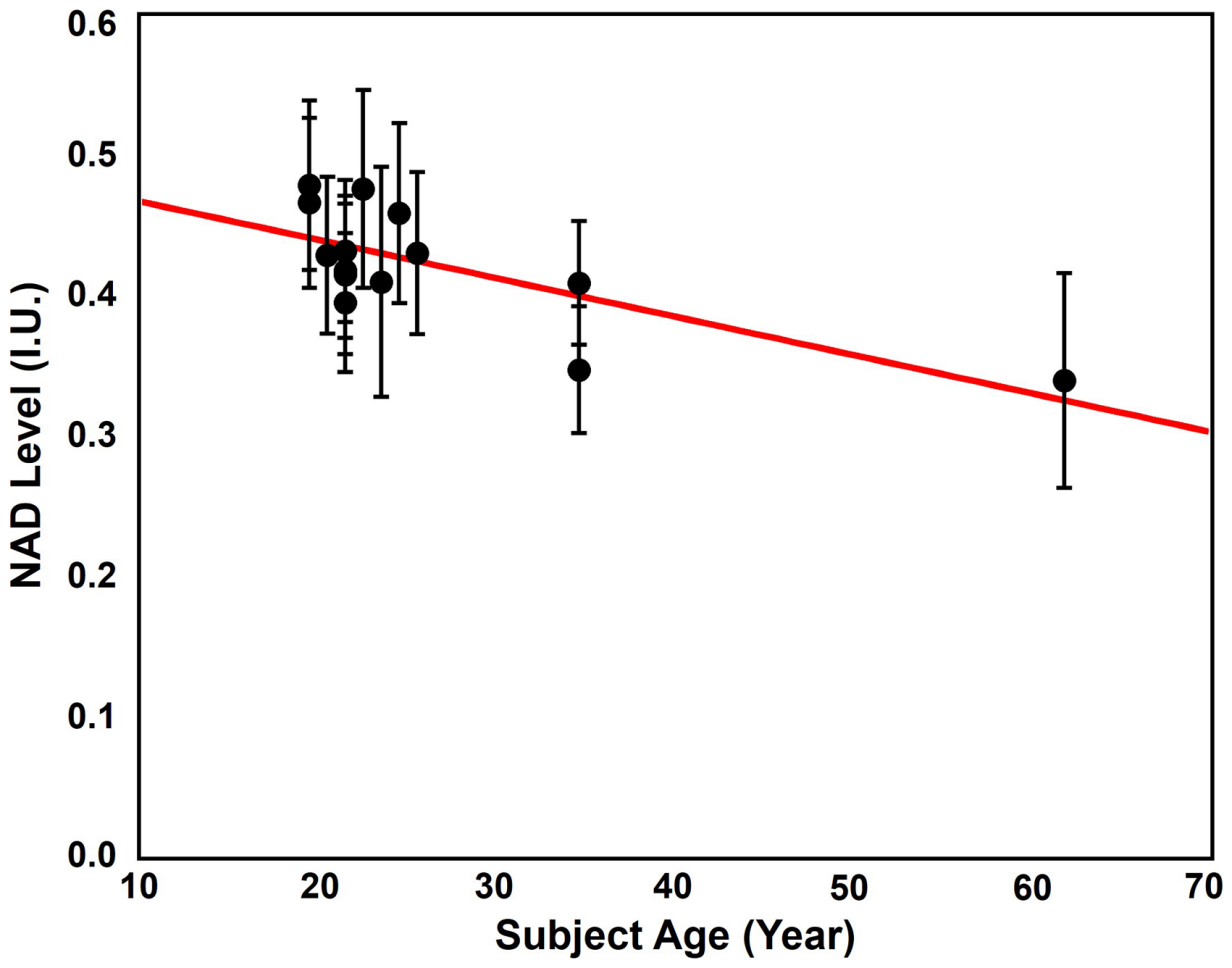
Correlation between the average brain NAD levels and age. The red line is the linear regression curve.

The age dependence of NAD level was revealed using the “1.0-cc data.” The NAD levels, which were signal intensities of NAD over the brain, of the 14 subjects and their ages were displayed in Figure 8. As we can see, cluster of the data with ages below 30 showed the highest overall NAD levels, and the NAD levels declined with ages above 30. The linear regression curve also confirmed this trend of NAD decline as age increases. The whole brain averaged spectra before and after denoising of these subjects were displayed in Supplementary Figure S5. This NAD decline with aging matched the biological expectation (Zhu et al., 2015), and could serve as strong indirect evidence to further support the feasibility of our ^31^P-MRSI method in mapping brain NAD content.

## 4 Discussion

Using ^31^P-MRSI techniques, the feasibility for mapping phosphorus metabolite distributions over the human brain with reasonable sensitivity and resolution has been demonstrated previously (Korzowski and Bachert, 2018; Korzowski et al., 2020, 2021; Rowland et al., 2020; Ruhm et al., 2021). However, for low-concentration metabolite signals such as NAD, it is still very challenging to measure and quantify due to the limited SNR in individual voxels. In this study, we applied a probabilistic subspace-based approach to process the ^31^P-MRSI data acquired at ultrahigh field and achieved significant noise reduction, which made it possible to reliably map and quantify the low-level NAD content across the entire human brain at 7 Tesla. Computational simulation and *in vivo* experiments were carried out to evaluate the SNR performance, accuracy, and reproducibility of the imaging method in NAD measurement. From the *in vivo* results, brain NAD concentrations around 0.4 mM were consistently found among multiple subjects. This averaged NAD level over the brain is in good agreement with the reported values obtained from human occipital and other brain regions (Zhu et al., 2015; Ruhm et al., 2021).

This feasibility study showed a relatively homogenous distribution of NAD levels over the brain. On the one hand, in the “2.3-cc data,” the spatial resolution is limited. Even though no hamming window was applied in processing to preserve spatial resolutions, partial volume effects could still partly contribute to the homogenous distribution we observed. On the other hand, the contrast analysis on the “1.0-cc data” also implied an insignificant NAD contrast between gray matter and white matter, especially compared with PCr and GPC. Even though only a small cohort was included in this analysis and further studies will be needed to confirm, the experimental evidence on the current data we have up to now consistently suggested such a homogenous distribution of brain NAD concentrations. This homogenous distribution of NAD might indicate a potentially uniform need for maintaining NAD homeostasis across the brain as ATP does, and might suggest that NAD plays a consistent role in supporting cellular functions throughout the entire brain.

This study showed a noticeable decline of brain NAD levels with aging (see Figure 8). This trend aligned with previous *ex vivo* studies and *in vivo* reports focusing on occipital and frontal lobes (Gomes et al., 2013; Zhu et al., 2015; Kim et al., 2017), suggesting a decline in mitochondrial metabolism efficiency and functionality during aging process. Successful reveal on the age dependence of NAD supported the feasibility of our method in measuring NAD changes under different biological conditions, which may have many potential applications in neurodegenerative diseases and aging-related disorders. But one limitation of this set of data is the limited size of cohort. Only 14 data were included for now and only three of them have an age above 30. In the future, including more subjects, especially more elderly volunteers, will strengthen the observations and evidence. In addition, more data in a wider age range may make it possible to understand better the age dependency of NAD concentration, even its variations in different age stages. It should also be noted that only the signal intensities after spectral fitting instead of quantified NAD concentrations were analyzed for the “1.0-cc data.” This is because NOE was employed in this dataset, which made the absolute quantification of metabolites challenging. In order to achieve absolute quantification, additional calibration scans and systematic studies will be needed, which is beyond the scope of this paper.

The partial separability model-based method has been widely used for noise reduction in not only ^31^P-MRSI, but also MRSI of other nuclei including ^1^H, ^13^C, ^2^H, etc. (Liang, 2007; Nguyen et al., 2013; Lam and Liang, 2014; Li et al., 2017; Guo et al., 2019). Compared with the basic low-rank approximation method used in previous ^31^P-MRSI studies (Korzowski et al., 2020; Ruhm et al., 2021), the probabilistic subspace method used in this work has several key features for enhanced noise reduction. First, the presented method pre-determined the temporal basis functions from the group data, in contrast to the low-rank approximation method which only depends on a single noisy data. One set of representative spectra of these basis functions was displayed in Supplementary Figure S4. This strategy leverages signal correlations across all the collected data to improve the SNR for subspace estimation, thus improving accuracy. Second, the presented method imposed two additional constraints on the spatial coefficients, including a total variation regularization and a statistical distribution constraint. The spatial regularization imposes anatomically weighted spatial smoothness on the coefficients, which has been widely used in various imaging reconstruction applications (Haldar et al., 2008; Lam and Liang, 2014; Zhao et al., 2015; Liu et al., 2018; Wang et al., 2020). The statistical distributions absorb prior information from the group data, which provide soft boundary constraints on the spatial coefficients to prevent them from going outside of the feasible distributions due to noise. These statistical constraints have been applied and proved effective for reliable estimations of myelin water components and B_1_ (radiofrequency magnetic field) mapping (Li et al., 2021a; Zhang et al., 2023).

**Figure 9:**
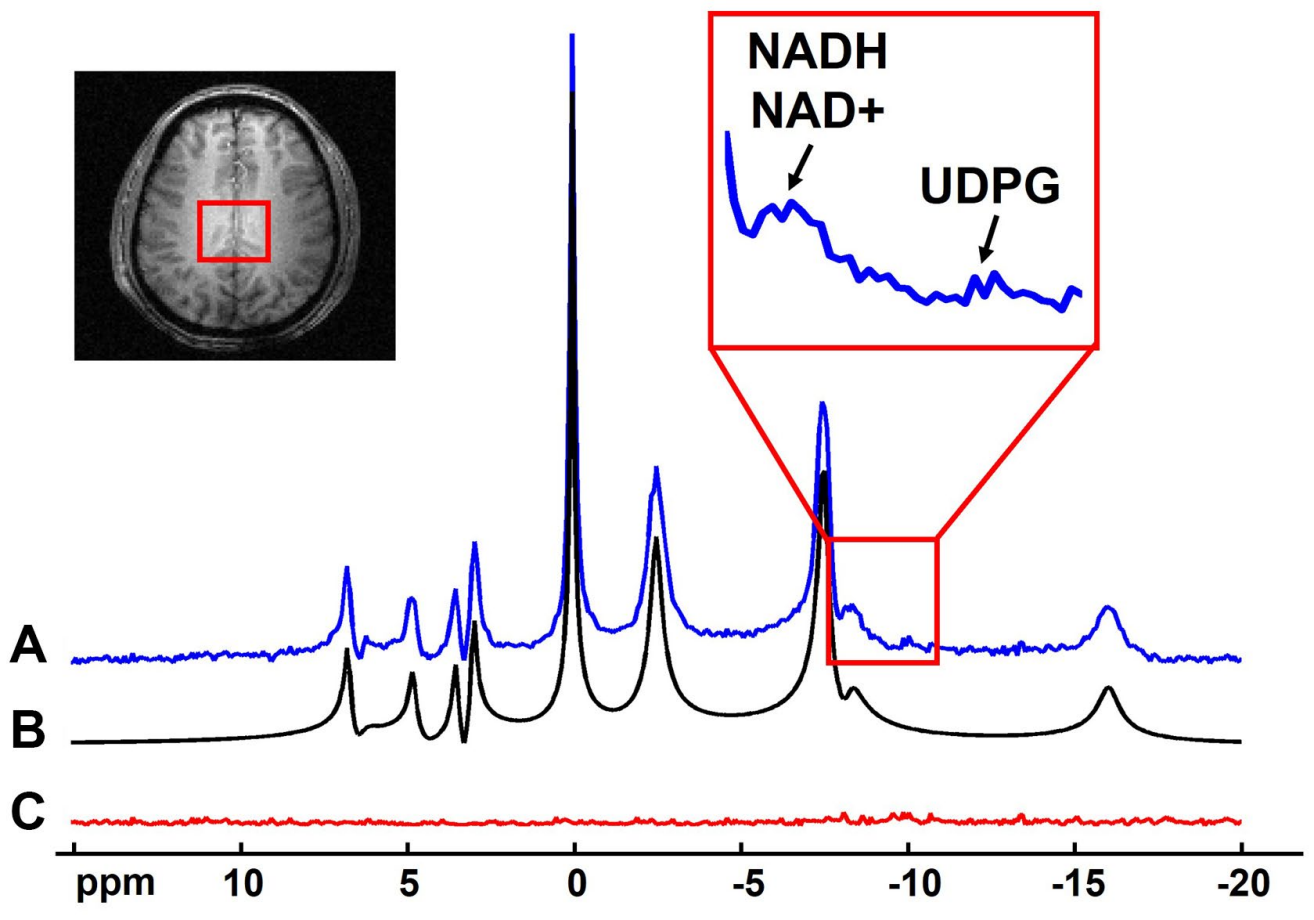
Representative spectrum (averaged from 9 adjacent voxels as indicated in the anatomical image, displayed in absolute mode) **(A)** with its spectral fitting **(B)** and residue **(C)**. Zoom-in region shows the spectral range covering the NADH/NAD^+^ and UDPG signals.

The noise reduction provided by the probabilistic subspace model can be utilized in many applications of ^31^P-MRSI techniques. On the one hand, the SNR enhancement can benefit the imaging resolution and/or speed, especially for those metabolites with relatively high concentrations (e.g., >1 mM). The “1.0-cc data” in this study has shown the potential in increasing spatial resolution, even for low-concentration metabolites like NAD. For applications where high-concentration metabolites are of interest or high resolutions are not required, the scan time can be significantly reduced, possibly to the level close to typical MR imaging scans. On the other hand, the SNR benefits can be applied to measure metabolites with even lower concentrations. For example, a spectrum averaging 9 adjacent voxels in the “2.3-cc data” is displayed in Figure 9. With the improved SNR, the doublet of NAD^+^ (which should be a doublet of doublet, but it is only visible as doublet in *in vivo* data due to wide linewidth) plus the singlet of NADH result in an apparent triplet, interestingly, similar with the ^31^P MRS acquired in the human occipital lobe with ^1^H-decoupling at 4 Tesla (Lu et al., 2014b). This result indicates great promise to separate these two metabolites on the voxel base, thus, to estimate and imaging the NAD redox ratio (=[NAD^+^]/[NADH]) in human brain (Lu et al., 2014b). Moreover, the Uridine Diphosphate Glucose (UDPG) resonance peaks at around −9.8 ppm were also above the noise level, whose concentrations were reported as around 0.1 mM (Ren et al., 2015). These observations demonstrated the potential of mapping the [NAD^+^]/[NADH] ratio and/or [UDPG] in the entire brain, possibly with a relatively lower resolution. This is one important direction of our future work.

**Figure 10:**
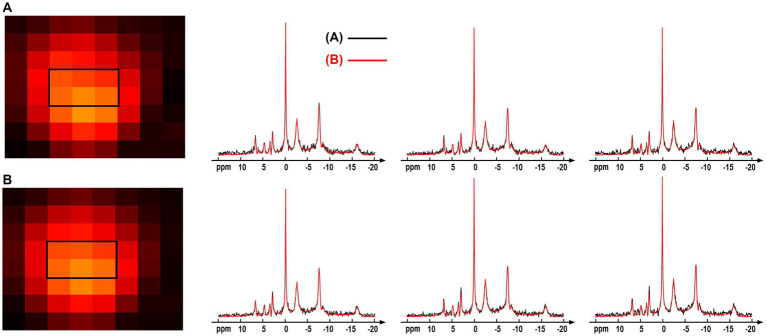
Comparison of the spectra and NAD estimates from **(A)** large-voxel data by averaging 24 voxels from the raw ^31^P-MRSI data; **(B)** large-voxel data by averaging 24 voxels from the denoised ^31^P-MRSI data. The spectra were from six representative large voxels indicated by black box in the NAD maps, and they were displayed in absolute mode.

The computation simulation study in Figure 3 used a group averaged ^31^P-MRSI data as the ground truth. This provided a good reference with realistic spectral lineshapes and allowed for quantitative evaluation on the performance of different processing methods. But using group-averaged spectra could potentially average out subtle variations of individual data. Therefore, we performed an additional comparison using the individual data. Specifically, one of the raw “2.3-cc data” was used, and every 24 voxels of the data were averaged to generate a large-voxel data with a relatively good SNR in each voxel. The proposed denoising method was applied on the raw small-voxel data and the same voxel-averaging was performed to generate the large-voxel data for comparison. The NAD estimates and localized spectra from these two data are shown in Figure 10. The results were reasonably consistent between the large-voxel raw data and denoised data, and the rRMSE on NAD estimates was 5.7%. This also supports the feasibility of the denoising method.

There are several potential improvements that can be made to achieve a more accurate quantification of NAD concentration. First, the excitation RF profile has not been corrected in the NAD quantification due to its small impact. For the “2.3 cc” data, the excitation hard pulse was centered in the middle of the αATP and γATP peaks. Therefore, the correcting factors of excitation profile for γATP and NAD are 1.038 and 1.071, respectively. Correcting this factor could slightly improve accuracy of NAD quantification. Second, the current spectral quantification model assumed a singlet resonance for the NAD peak, while the NAD peak should include NAD^+^, NADH, and several UDPG components (Ren et al., 2020; Ren and Sherry, 2021). Separating these components requires exceptional SNR and more complicated signal modeling. Being able to separate these components will further improve the accuracy of NAD quantification.

The technical advancements in MR hardware and systems could further improve the sensitivity of such ^31^P-MRSI methods. The ultrahigh field systems used in this study were 7 T scanners, while there are several MR human systems with higher field strength, such as 9.4 and 10.5 T. Higher field strength could further boost the SNR, thus enhancing the imaging capability of ^31^P-MRSI techniques. We used volume ^31^P RF coils in this study. It is well known that ^31^P RF array coil with multiple receive channels could provide better SNR than the volume coil (van Uden et al., 2019; Rowland et al., 2020; Peeters et al., 2021). Combined with a higher field strength, better RF coils, and the advanced processing methods used in this work, the ^31^P-MRSI technology has the potential to provide sufficient resolution and speed for practical applications in research and clinical settings.

The presented capability of non-invasively mapping NAD levels in entire human brain is expected to be highly valuable and have broad implications in many biomedical and clinical research fields. It will also allow evaluation of the alteration of NAD levels in diseased brain regions due to neurological disorders, neurodegenerative diseases, or age-related cognitive decline. Mapping NAD contents in these conditions may uncover specific patterns of regional NAD-related metabolic dysregulation associated with disease progression, potentially opening avenues for targeted interventions and therapeutic strategies. In addition to its impact on disease research, this technique could serve as a powerful imaging tool for neurodevelopmental studies, which allows for the exploration of how brain NAD dynamics contribute to the maturation and developmental progression of the brain.

## 5 Conclusion

In conclusion, by combining the ultrahigh field 3D ^31^P-MRSI with advanced probabilistic subspace-model based post-processing method, we demonstrated for the first time the feasibility of whole-brain NAD mapping in healthy human subjects at 7 T. This approach would allow non-invasively monitoring cerebral NAD and its changes in brain regions under various brain conditions.

## Data availability statement

The raw data supporting the conclusions of this article will be made available by the authors, without undue reservation.

## Ethics statement

The studies involving humans were approved by the Institutional Review Board of the University of Minnesota; Institutional Review Board at the University of Pittsburgh. The studies were conducted in accordance with the local legislation and institutional requirements. The participants provided their written informed consent to participate in this study.

## Author contributions

RG: Formal analysis, Investigation, Methodology, Writing – original draft, Writing – review & editing. SY: Data curation, Formal analysis, Writing – review & editing. HW: Data curation, Investigation, Writing – review & editing. YL: Conceptualization, Formal analysis, Methodology, Writing – review & editing. YZ: Data curation, Methodology, Writing – review & editing. Z-PL: Conceptualization, Investigation, Resources, Supervision, Writing – review & editing. WC: Conceptualization, Formal analysis, Project administration, Resources, Supervision, Validation, Writing – review & editing. X-HZ: Conceptualization, Data curation, Funding acquisition, Resources, Supervision, Validation, Writing – review & editing.
