# Supplementary material for: Mapping intracellular NAD content in entire human brain using phosphorus-31 MR spectroscopic imaging at 7 Tesla
Source: Front Neurosci. 2024 Jun 7;18:1389111. doi: 10.3389/fnins.2024.1389111 (PMC11190064; doi:10.3389/fnins.2024.1389111)
Supplement: Supplementary file 1 [file Data_Sheet_1.docx]

Supplementary Material

# Supplementary Figures and Tables

## Supplementary Figure:


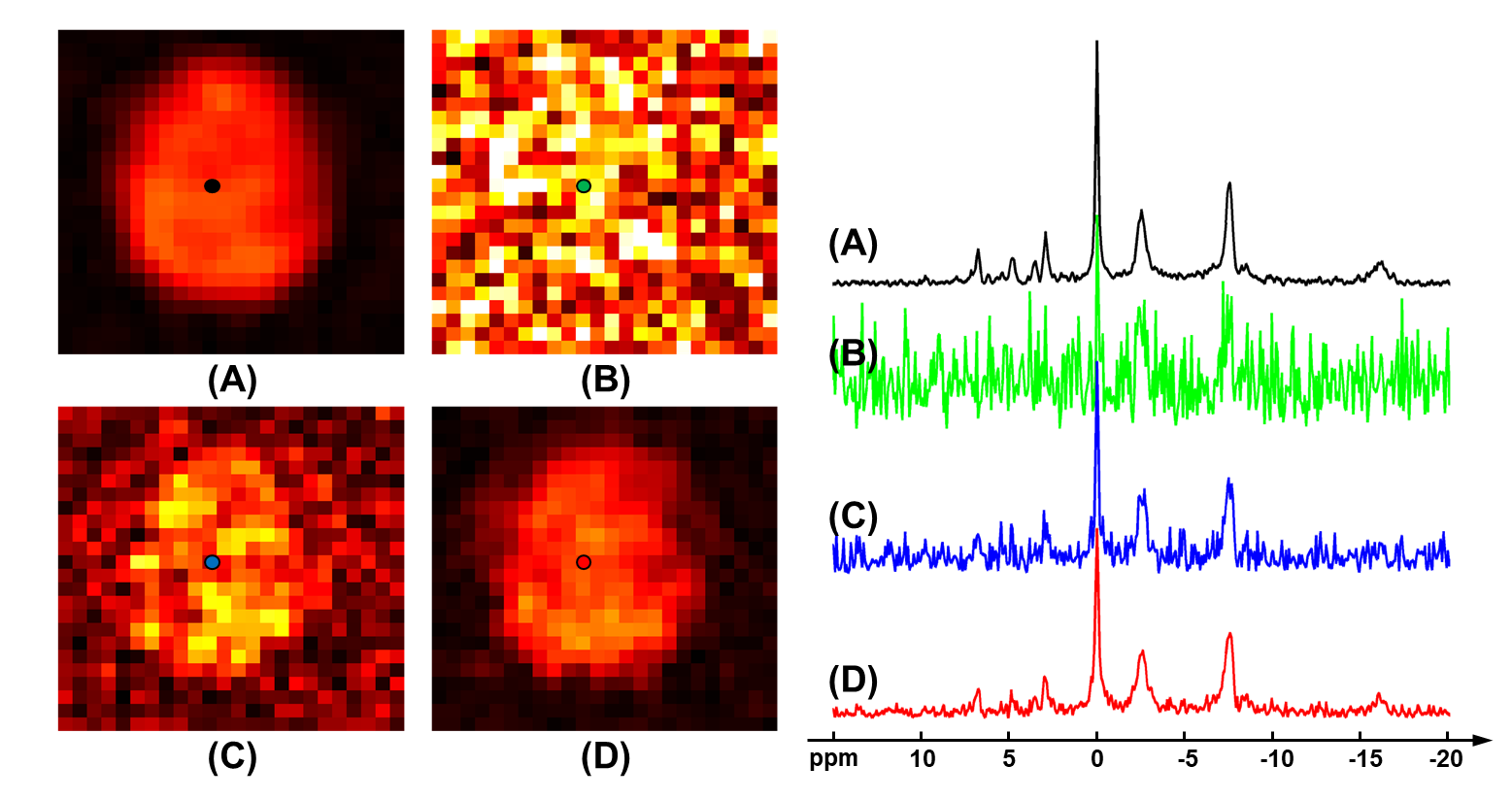


**Supplementary Figure S1.** Computational simulation of ^31^P-MRSI signals with a similar noise level of the “1.0 cc data”. Similar to **Figure 3**, the comparison of different methods was displayed: (**A**) ground truth; (**B**) raw data without denoising; (**C**) basic low-rank denoising; (**D**) probabilistic subspace-based denoising method. NAD maps (signal intensities after spectral fitting) were displayed on the left and the localized spectra of selected points were displayed on the right. The rRMSEs (relative root-mean-square-errors) were 117.9%, 42.5%, and 18.2% for (**B**), (**C**), and (**D**), respectively. The spectra were displayed in absolute mode.


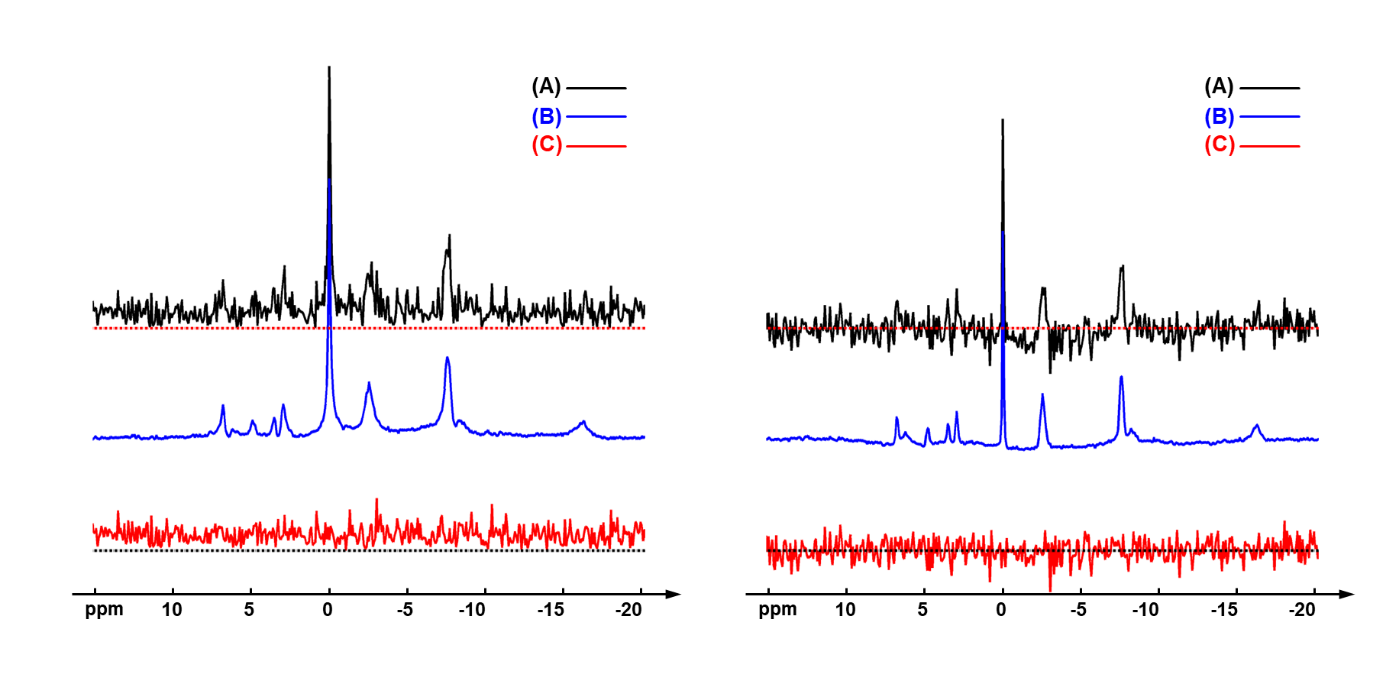


**Supplementary Figure S2**. The raw spectrum (A), denoised spectrum (B), and their difference, the noise spectrum (C) of the example spectra shown in Figure 1 of the manuscript. On the left, these spectra are displayed in absolute mode; on the right, these spectra are displayed in real mode (extracted after zero-order and first-order phase correction). The dash lines are zero baselines.


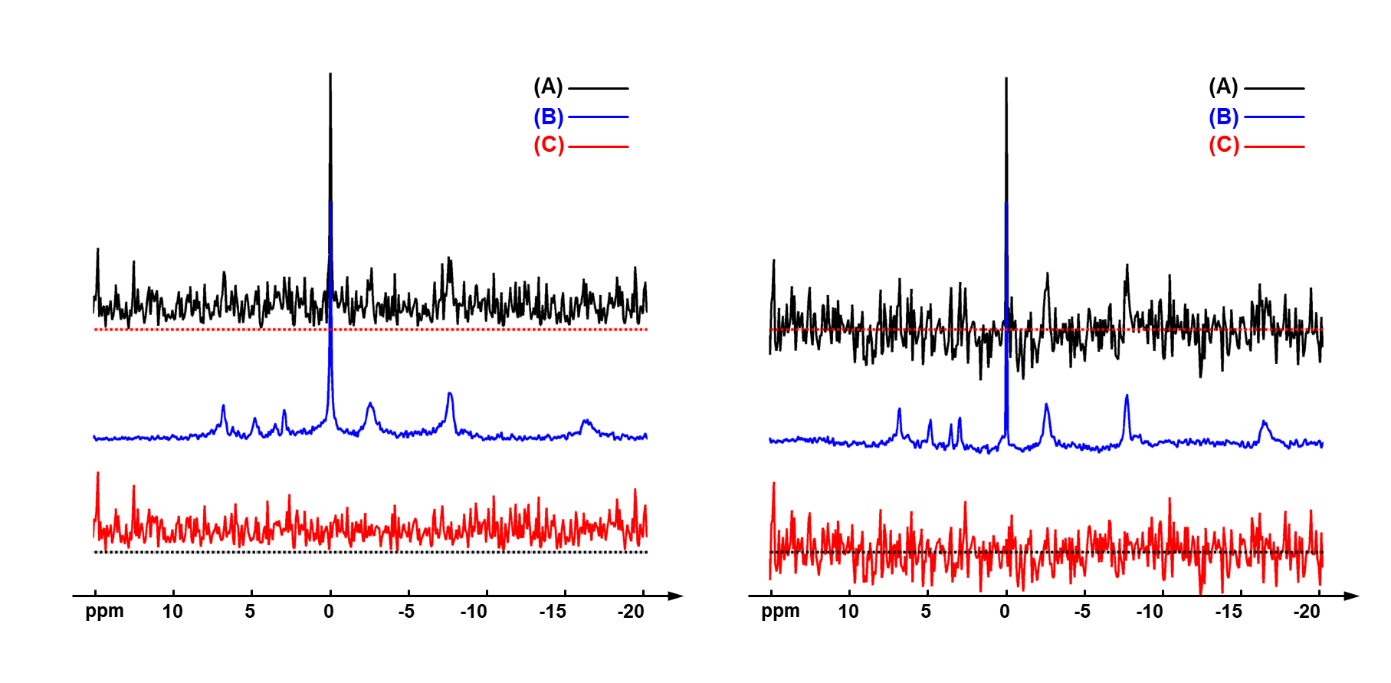


**Supplementary Figure S3**. The raw spectrum (A), denoised spectrum (B), and their difference, the noise spectrum (C) of the example spectra shown in Figure 5 of the manuscript. On the left, these spectra are displayed in absolute mode; on the right, these spectra are displayed in real mode (extracted after zero-order and first-order phase correction). The dash lines are zero baselines.


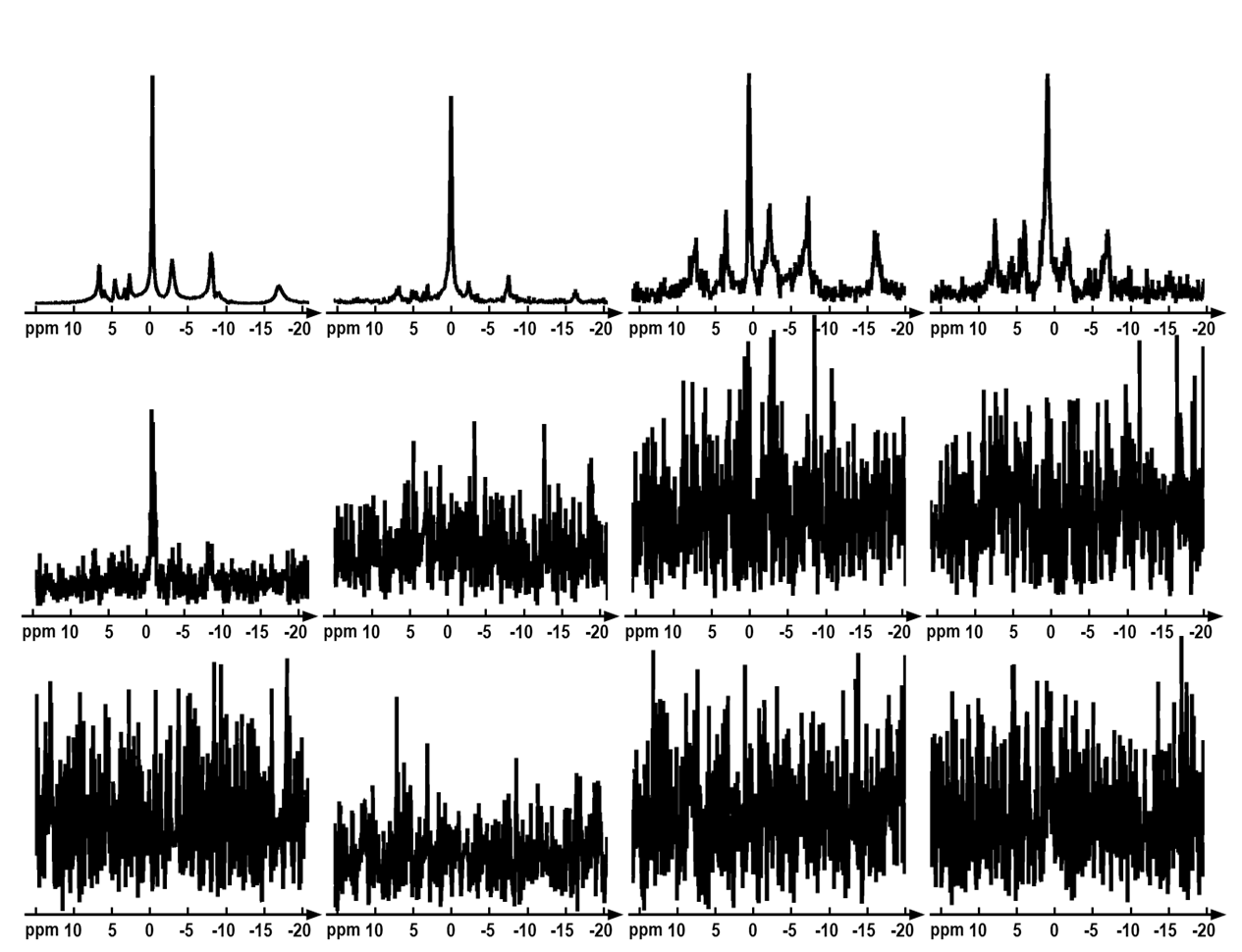


**Supplementary Figure S4**. The spectra of the first 12 components of basis functions derived from the group “1.0 cc data”. These components were displayed on scales by their own maximal values. This order number 12 was chosen based on the distributions of the singular values of the Casorati matrix. The spectra were displayed in absolute mode.


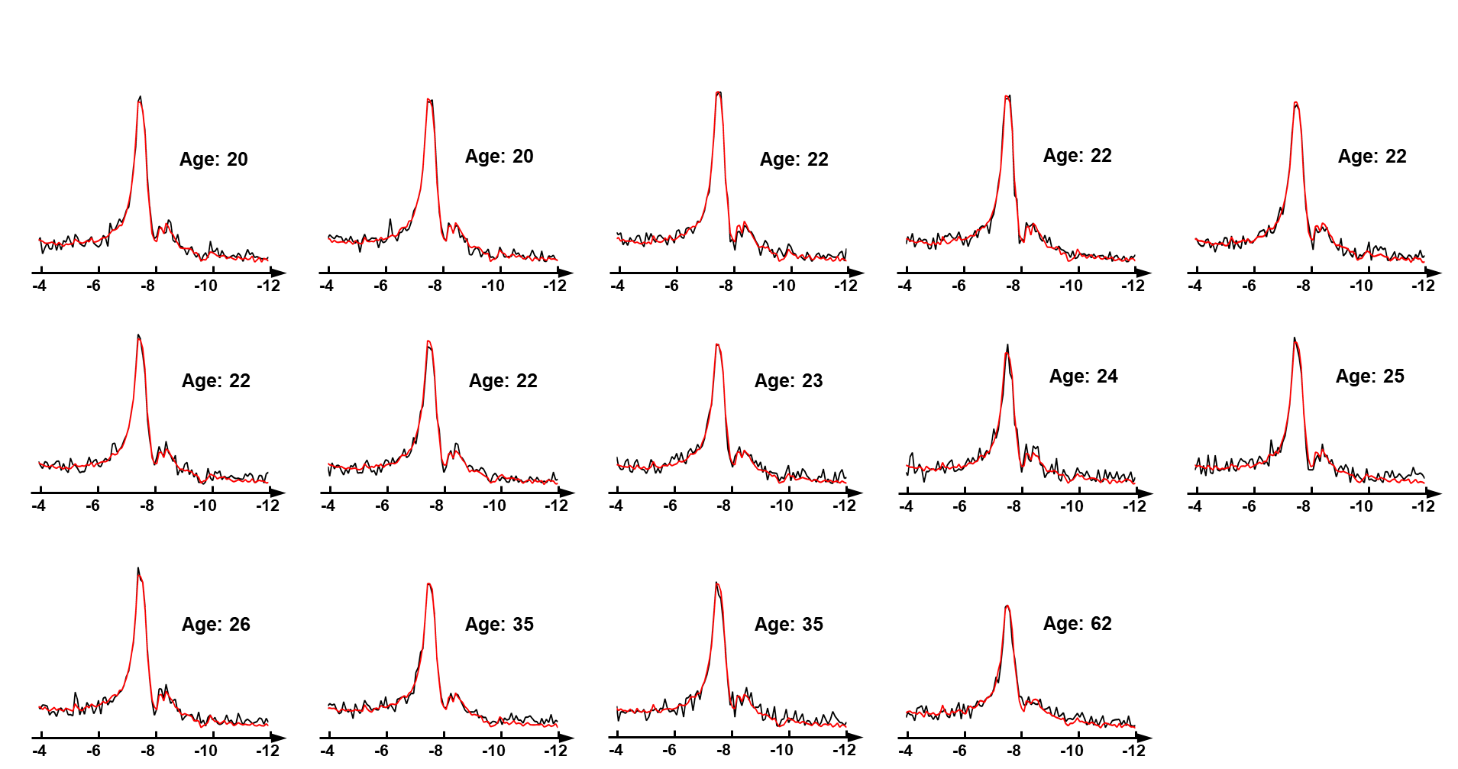


**Supplementary Figure S5**. The whole brain averaged spectra in the NAD chemical shift region from all fourteen subjects in the “1.0 cc data”. The spectra were displayed in absolute mode.

**
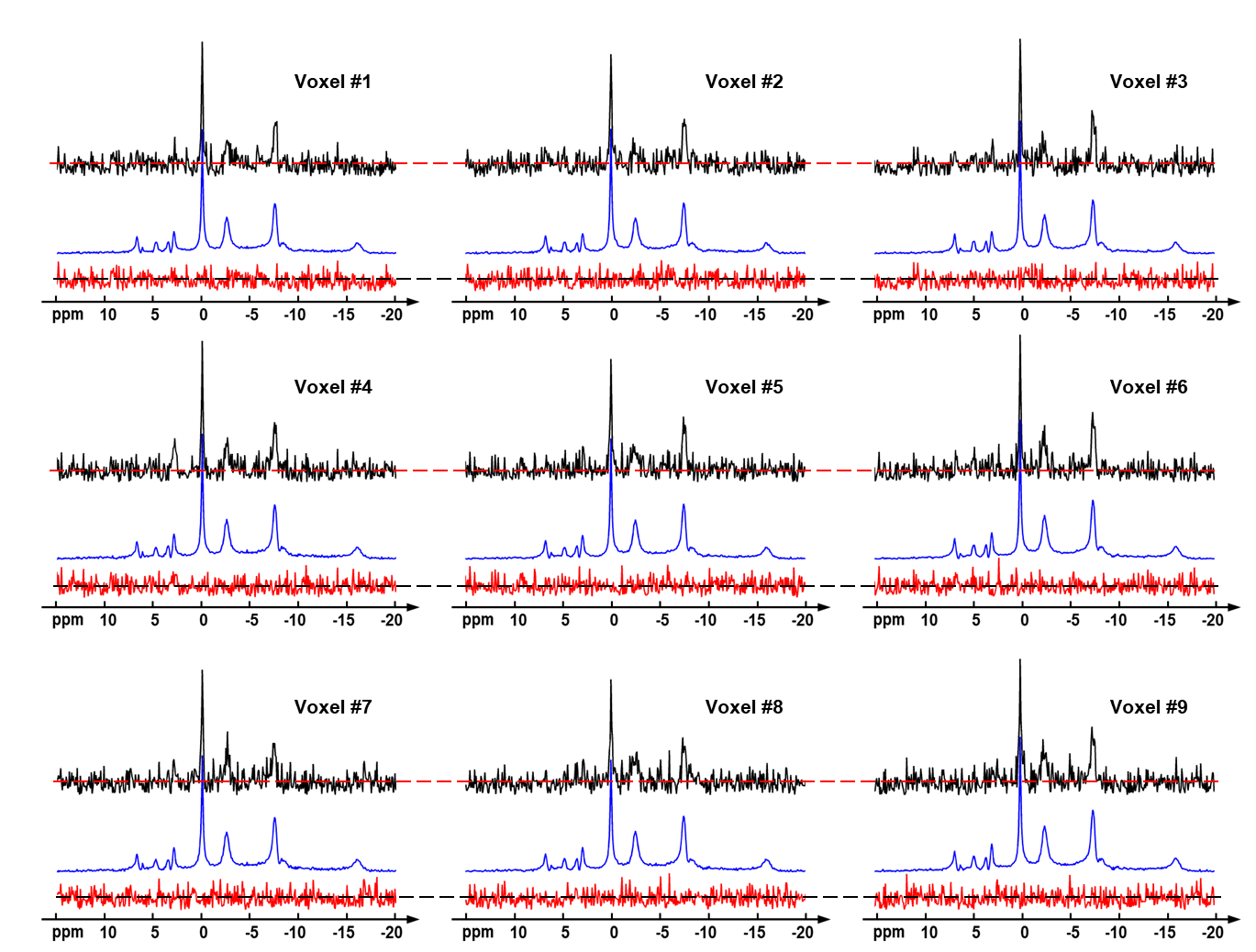
**

**Supplementary Figure S6**. The raw spectra (in black), denoised spectra (in blue), and removed noise (in red) of the nine voxels in Figure 9. The spectra were displayed in absolute mode, the dash lines are the centers of noise spectra.

Supplementary Tables:

**Supplementary Table S1.** Summary of hardware used in this work according to Minimum Reporting Standards for *in vivo* Magnetic Resonance Spectroscopy (MRSinMRS).

| **1. Hardware** | | |
| --- | --- | --- |
| Site | University of Minnesota  (2.3 cc data) | University of Pittsburgh  (1.0 cc data) |
| a. Field strength [T] | 7 T | 7 T |
| b. Manufacturer | Magnex Scientific, Abingdon, U.K. | Siemens Healthineers, Erlangen, Germany |
| c. Model (software version if available) | With a Varian INOVA console | Magnetom 7 T (VB17) |
| d. RF coils: nuclei (transmit/receive), number of channels, type, body part | ^31^P/^1^H dual-tuned TEM head volume coil | ^31^P/^1^H dual-tuned birdcage head volume coil (Rapid Biomedical, Rimpar, Germany) |
| e. Additional hardware | N/A | N/A |

**Supplementary Table S2.** Summary of acquisition sequences used in this work according to Minimum Reporting Standards for in vivo Magnetic Resonance Spectroscopy (MRSinMRS).

| **2. Acquisition** | | |
| --- | --- | --- |
| Site | University of Minnesota  (2.3 cc data) | University of Pittsburgh  (1.0 cc data) |
| a. Pulse sequence | 3D CSI-FID | 3D CSI-FID |
| b. Volume of interest (VOI) locations | Covering whole brain | Covering whole brain |
| c. Nominal VOI size | 20×20×22 cm^3^ | 22×22×10 cm^3^ |
| d. Repetition time (TR), echo time (TE) [ms, s] | TR/TE = 500/1.0 ms, | TR/TE = 200/1.0 ms |
| e. Total number of excitations or acquisitions per spectrum | 40 averages (weighted) | 18 averages (weighted) |
| f. Additional sequence parameters | Excitation pulse: 0.5 ms rectangular pulse.  Flip angle = 45°  Readout bandwidth = 5 kHz  Vector size = 512  No acceleration factor  Weighted sampling using Fourier series window to generate cylindrical voxels with circular shape in plane and rectangular in the 3rd dimension.  No NOE was applied | Excitation pulse: 1.2 ms SINC pulse with 5.1 kHz bandwidth.  Flip angle = 30°  Readout bandwidth = 5 kHz  Vector size = 512  No acceleration factor  Weighted sampling  NOE was applied (two 90° hard pulses with 5.0 ms pulse duration and 10.0 ms inter-pulse duration; B_1_ values were 1.2 uT using 160V reference voltage) |
| g. Water suppression method | NA | NA |
| h. Shimming method, reference peak, and thresholds for “acceptance of shim” chosen | Automated 3D B_0_ field mapping technique followed by manual adjustment. | Automated 3D B_0_ field mapping technique followed by manual adjustment. |
| i. Triggering or motion correction method | NA | NA |

**Supplementary Table S3.** Summary of acquisition sequences used in this work according to Minimum Reporting Standards for in vivo Magnetic Resonance Spectroscopy (MRSinMRS).

| **3. Data analysis methods and outputs** | | |
| --- | --- | --- |
| Site | University of Minnesota  (2.3 cc data) | University of Pittsburgh  (1.0 cc data) |
| a. Analysis software | In-house developed MATLAB scripts | In-house developed MATLAB scripts |
| b. Processing steps deviating from quoted reference or product | Spectral alignment, denoising and then quantification as descripted in the manuscript | Spectral alignment, denoising and then quantification as descripted in the manuscript |
| c. Output measure (e.g., absolute concentration, institutional units, ratio) | Both institutional units  and absolute concentration | Institutional units |
| d. Quantification references and assumptions, fitting model assumptions | γATP as an internal reference with a concentration of 2.8 mM;  Fitting model assumed signals of ^31^P metabolites were singlet resonances with Gaussian line shape. | γATP as an internal reference, but no absolute value was assigned due to NOE;  Fitting model assumed signals of ^31^P metabolites were singlet resonances with Gaussian line shape. |
